# Supplementary material for: DFT Study of the Direct Radical Scavenging Potency of Two Natural Catecholic Compounds
Source: Int J Mol Sci. 2022 Nov 21;23(22):14497. doi: 10.3390/ijms232214497 (PMC9697371; doi:10.3390/ijms232214497)
Supplement: Supplementary file 1 [file ijms-23-14497-s001.zip › ijms-2030804-supplementary.pdf]

SUPPLEMENTARY MATERIALS

Chart S1.

Graphically presented correlations between thermodynamic and kinetic parameters for reaction of quercetin with $CH_3OO^{\bullet}$ in pentyl ethanoate.

path	BDE kcal/mol	$\Delta_r G$ kcal/mol	ν cm^{-1}	$\Delta G^{\neq}$ kcal/mol	k^{TST} M^{-1} s^{-1}	$k^{TST/Eck}$ M^{-1} s^{-1}	$k^{TST/Wig}$ M^{-1} s^{-1}
C-3	85.41	0.9	-3307	22.7	1.4×10^{-4}	2.6×10^{0}	1.6×10^{-3}
C-5	99.41	14.9	-2631	30.6	2.5×10^{-10}	3.0×10^{-8}	1.9×10^{-9}
C-7	94.55	10.4	-2456	23.3	4.9×10^{-5}	6.4×10^{-4}	3.3×10^{-4}
C-3'	82.36	-1.1	-2227	16.3	7.2×10^{0}	4.7×10^{2}	4.2×10^{1}
C-4'	80.35	-3.5	-2259	16.5	4.7×10^{0}	3.6×10^{2}	2.8×10^{1}

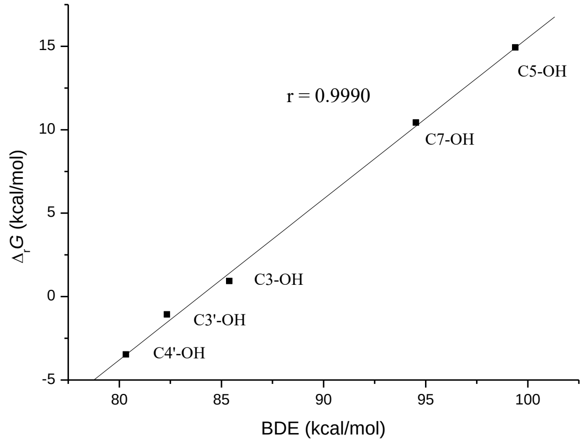

a) Plot of $\Delta_r G$ *vs* the O−H BDE.

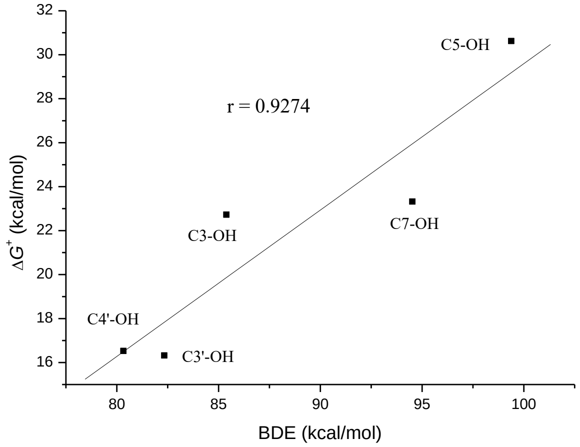

b) Plot of $\Delta G^{\neq}$ *vs* the O−H BDE.

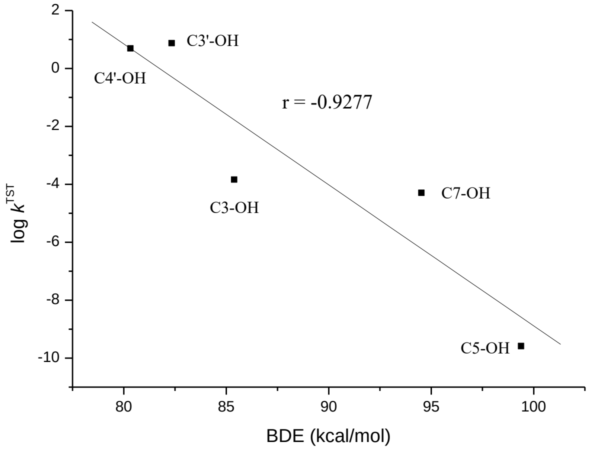

c) Plot of log k^{TST} *vs* the O−H BDE.

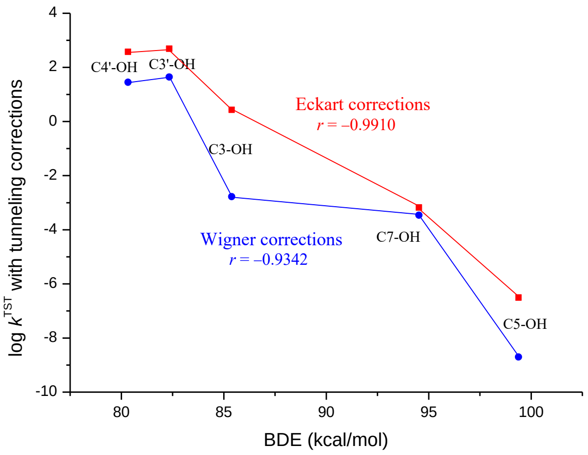

d) Plot of log $k^{TST/Eck}$ ($k^{TST/Wig}$) *vs* the O−H BDE.

Chart S2.

Graphically presented correlations between thermodynamic and kinetic parameters for reaction of quercetin with $HOO^{\bullet}$ in pentyl ethanoate.

path	BDE kcal/mol	$\Delta_r G$ kcal/mol	ν cm^{-1}	$\Delta G^{\neq}$ kcal/mol	k^{TST} M^{-1} s^{-1}	$k^{TST/Eck}$ M^{-1} s^{-1}	$k^{TST/Wig}$ M^{-1} s^{-1}
C-3	85.41	-1.3	-4274	18.3	2.3×10^{-1}	2.4×10^{3}	4.3×10^{0}
C-5	99.41	12.7	-3894	27.2	7.8×10^{-8}	1.8×10^{-5}	1.2×10^{-6}
C-7	94.55	8.1	-2521	23.3	5.2×10^{-5}	5.0×10^{-3}	3.7×10^{-4}
C-3'	82.36	-3.4	-1884	16.3	7.6×10^{0}	2.8×10^{2}	3.4×10^{1}
C-4'	80.35	-5.8	-1843	16.4	6.1×10^{0}	2.1×10^{2}	2.6×10^{1}

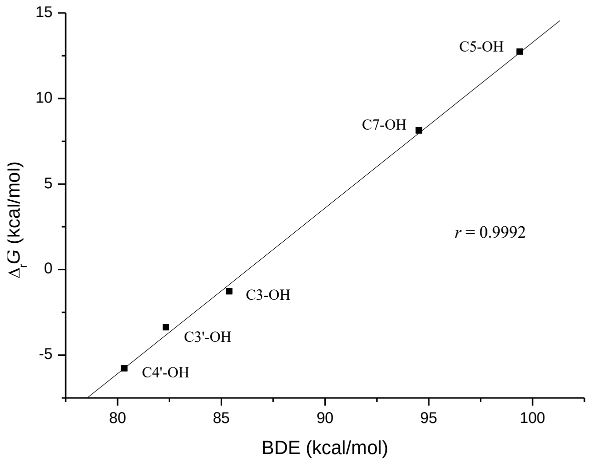

a) Plot of $\Delta_r G$ *vs* the O−H BDE.

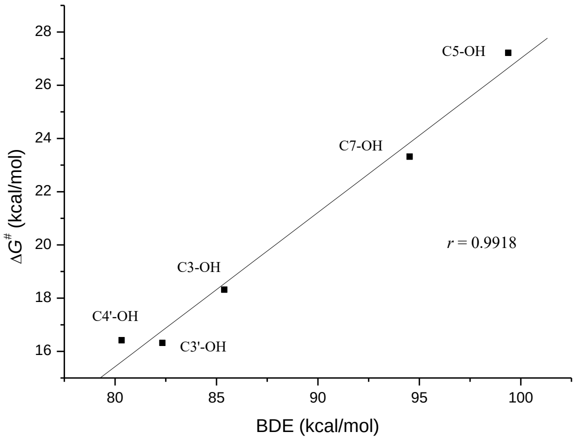

b) Plot of $\Delta G^{\neq}$ *vs* the O−H BDE.

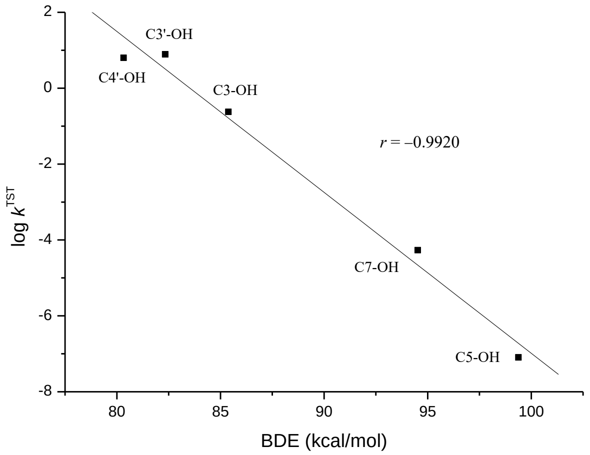

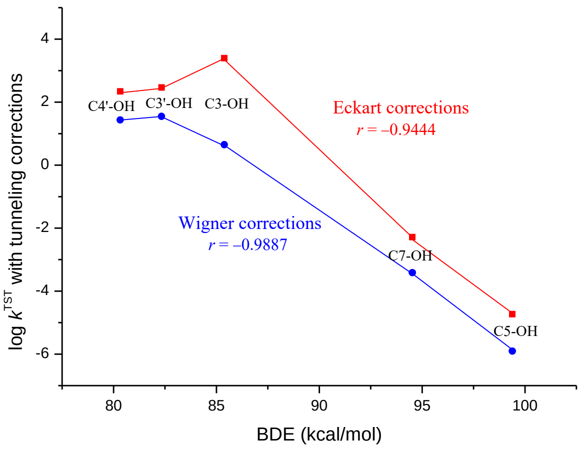

c) Plot of log k^{TST} *vs* the O−H BDE. d) Plot of log $k^{TST/Eck}$ ($k^{TST/Wig}$) *vs* the O−H BDE.

Chart S3.

Graphically presented correlations between thermodynamic and kinetic parameters for reaction of rooperol with $HOO^{\bullet}$ in pentyl ethanoate.

path	BDE kcal/mol	$\Delta_r G$ kcal/mol	ν cm^{-1}	$\Delta G^{\neq}$ kcal/mol	k^{TST} M^{-1} s^{-1}	$k^{TST/Eck}$ M^{-1} s^{-1}
3'-OH	80.48	-4.8	-1801	16.3	7.4×10^{0}	1.7×10^{2}
4'-OH	77.04	-8.0	-1616	14.7	9.7×10^{1}	1.1×10^{3}
3"-OH	81.81	-3.0	-1817	17.6	8.2×10^{-1}	2.5×10^{1}
4"-OH	79.63	-5.3	-1761	16.5	5.0×10^{0}	1.2×10^{2}
[a]allylic C-H	72.21	-14.1	-1739	19.4	3.6×10^{-2}	1.4×10^{0}
[b]allylic C-H	72.21	-14.1	-1730	19.3	4.3×10^{-2}	1.6×10^{0}

[a]allylic C-H in front of the molecular plane; [b]allylic C-H in the back of the molecular plane

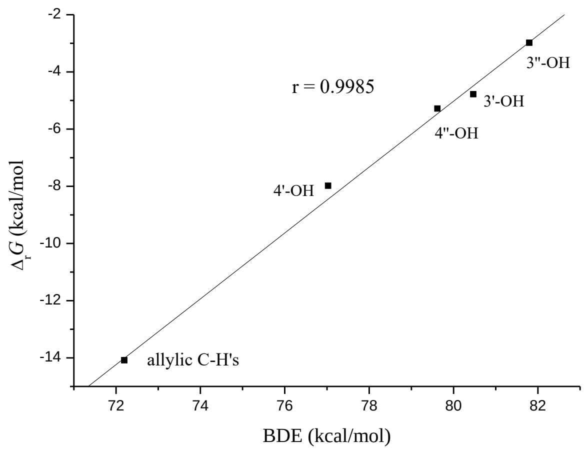

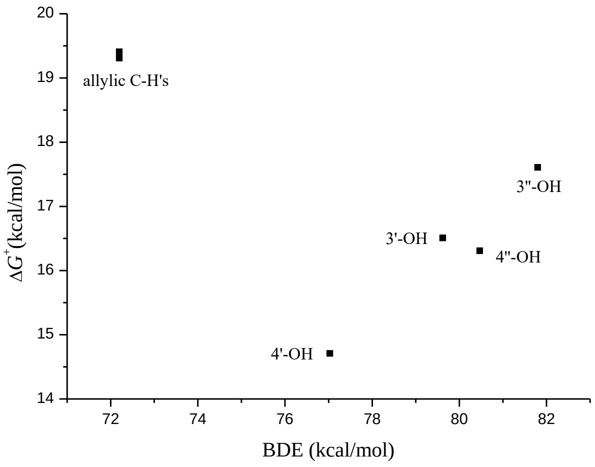

a) Plot of $\Delta_r G$ *vs* BDE. b) Plot of $\Delta G^{\neq}$ *vs* BDE.

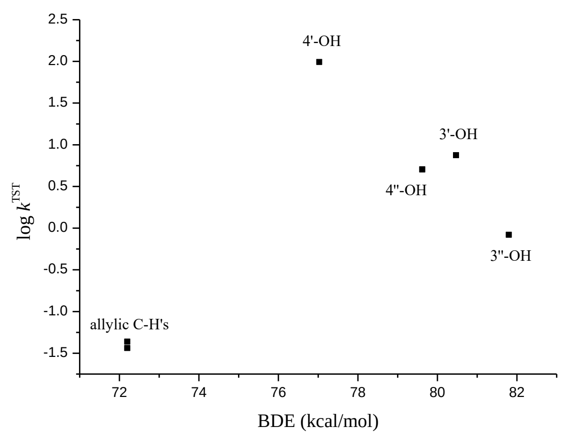

c) Plot of log k^{TST} *vs* BDE.

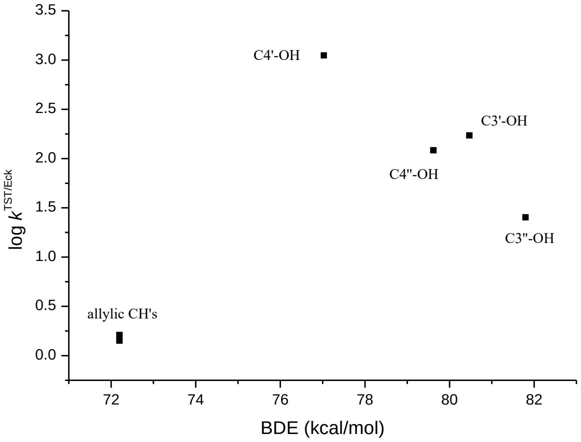

d) Plot of log $k^{TST/Eck}$ *vs* BDE.

Chart S4.

Graphically presented correlations between thermodynamic and kinetic parameters for reaction of rooperol with $CH_3OO^{\bullet}$ in pentyl ethanoate.[a]

path	BDE kcal/mol	$\Delta_r G$ kcal/mol	ν cm^{-1}	$\Delta G^{\neq}$ kcal/mol	k^{TST} M^{-1} s^{-1}	$k^{TST/Eck}$ M^{-1} s^{-1}
3'-OH	80.48	-2.6	-2085	17.1	1.7×10^{0}	7.8×10^{1}
4'-OH	77.04	-5.7	-1866	15.8	1.7×10^{1}	3.2×10^{2}
3''-OH	81.81	-0.7	-2087	17.7	6.7×10^{-1}	3.6×10^{1}
4''-OH	79.63	-3.0	-1992	17.2	1.5×10^{0}	5.5×10^{1}

[a] Allylic paths were not considered.

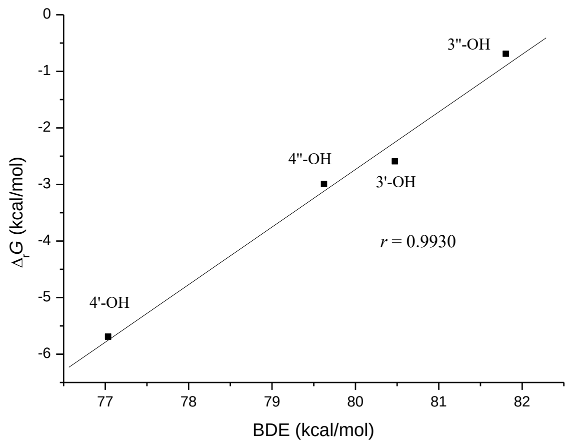

a) Plot of $\Delta_r G$ *vs* BDE.

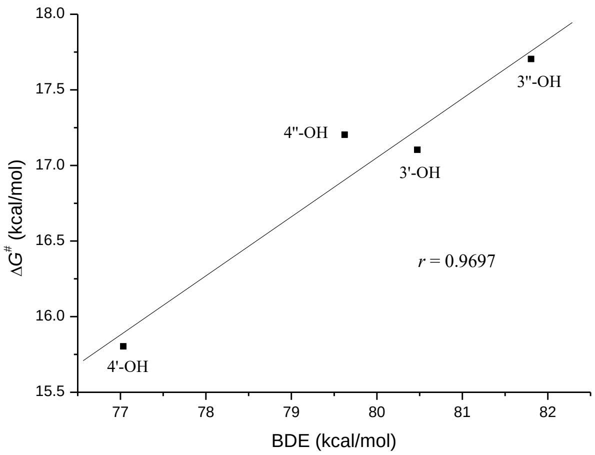

b) Plot of $\Delta G^{\neq}$ *vs* BDE.

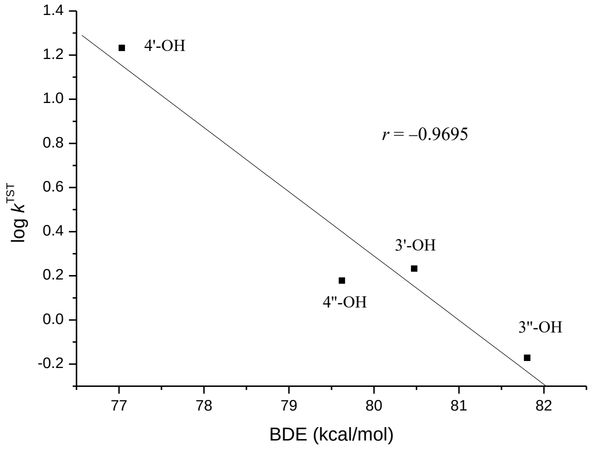

c) Plot of log k^{TST} *vs* BDE.

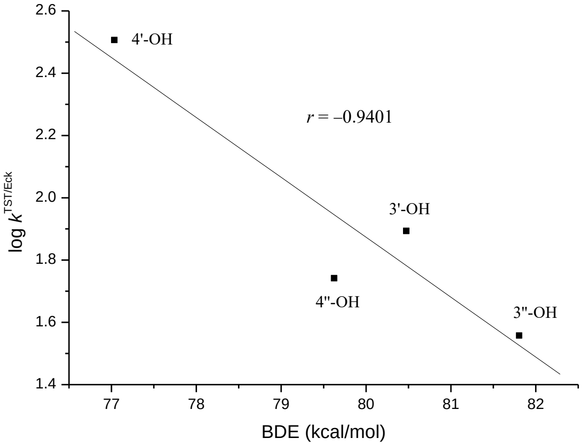

d) Plot of log $k^{\mathrm{TST/Eck}}$ *vs* BDE.

Table S1. Kinetic data obtained by taking into account reactant complex and product complex for the PCET paths of quercetin with $HOO^{\bullet}$ and $CH_3OO^{\bullet}$ radicals, in pentyl ethanoate at 298.15 K. $k_{\mathrm{overall}}^{\mathrm{TST/Eck}}$ is the sum of the rate constants of all reaction paths.[a]

path	BDE	$\Delta_r G$	ν	$\Delta G^{\neq}$	k^{TST}	κ^{Eck}	$k^{\mathrm{TST/Eck}}$	Γ
**$HOO^{\bullet}$**								
3-OH	85.41	-1.3	-4273.77	18.3	2.3×10^{-1}	7090769.7	1.6×10^{6}	100
5-OH	99.41	12.7	-3894.44	27.2	7.8×10^{-8}	318443.4	2.5×10^{-2}	0
7-OH	94.55	6.9	-2521.38	21.3	1.4×10^{-3}	1794.8	2.6×10^{0}	0
3'-OH	82.36	-3.4	-1883.66	16.3	7.6×10^{0}	84.8	6.4×10^{2}	0
4'-OH	80.35	-5.8	-1843.08	16.4	6.1×10^{0}	64.8	4.0×10^{2}	0
						$k_{\mathrm{overall}}^{\mathrm{TST/Eck}}$ =	1.6×10^{6}	
**$CH_3OO^{\bullet}$**								
3-OH	85.41	0.9	-3306.98	22.7	1.4×10^{-4}	198437.3	2.8×10^{1}	0.65
5-OH	99.41	14.9	-2631.22	30.6	2.5×10^{-10}	2359.1	5.8×10^{-7}	0
7-OH	94.55	10.4	-2455.59	23.3	4.9×10^{-5}	281.5	1.4×10^{-2}	0
3'-OH	82.36	-1.1	-2226.66	16.3	7.2×10^{0}	333.0	2.4×10^{3}	55.45
4'-OH	80.35	-3.5	-2259.20	16.5	4.7×10^{0}	411.2	1.9×10^{3}	43.90
						$k_{\mathrm{overall}}^{\mathrm{TST/Eck}}$ =	4.3×10^{3}	

[a] Wigners tunneling corrections remains the same as in Table 1.

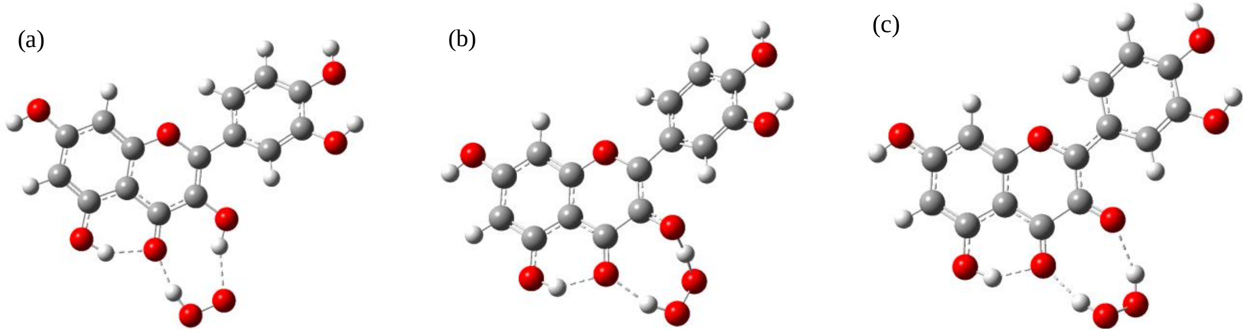

Optimized geometries in pentyl ethanoate obtained with the M05-2X/6-311++G(d,p) method for the reaction of $HOO^{\bullet}$ with 3-OH group of quercetin: (a) reactant complex (RC) – planar structure, (b) transition state (TS), and (c) product complex (PC).

RC, TS and PC are considered in the rate constant and Eckart tunneling estimation using Eyringpy program [a,b].
Only TS imaginary frequency $\nu\,(\mathrm{cm}^{-1})$ is considered in Wigner method. Consequently, Wigner tunneling corrections are the same, with or without RC and PC consideration.

[a] Dzib, E.; Cabellos, J.L.; Ortiz-Chi, F.; Pan, S.; Galano, A.; Merino, G. Eyringpy: A program for computing rate constants in the gas phase and in solution. *Int. J. Quantum Chem*. **2019**, *119*, e25686.
[b] Alvarez-Idaboy, J.R.; Mora-Diez, N.; Boyd, R.J.; Vivier-Bunge, A. On the importance of prereactive complexes in mole-cule-radical reactions: Hydrogen abstraction from aldehydes by OH. *J. Am. Chem. Soc*. **2001**, *123*, 2018–2024.

Chart S5.

Calculation of molar fractions (*f*) of quercetin species at pH = 7.4

$pK_{a1} = 6.41$; $pK_{a2} = 7.81$; $pK_{a3} = 10.19$

Acidity data taken from [c]:
[c] R. Alvarez-Diduk, M.T. Ramirez-Silva, A. Galano, A. Merkoci, Deprotonation mechanism and acidity constants in aqueous solution of flavonols: a combined experimental and theoretical study. *J. Phys. Chem. B* **2013**, *117*, 12347−12359.

Calculation of molar fractions was performed according to a study [d]:
[d] A. Galano, J.R. Alvarez-Idaboy, A computational methodology for accurate predictions of rate constants in solution: Application to the assessment of primary antioxidant activity, *J. Comput. Chem*. **2013**, *34*, 2430−2445.

$f[A^{3-}] = 1/(1 + \beta_1\,[H^+] + \beta_2\,[H^+]^2 + \beta_3\,[H^+]^3)$

At pH = 7.4, $[H^+] = 3.98 \times 10^{-8}$ M

$\beta_1 = 10^{pKa3} = 10^{10.19}$

$\beta_2 = 10^{pKa3 + pKa2} = 10^{10.19 + 7.81} = 10^{18}$

$\beta_3 = 10^{pKa3 + pKa2 + pKa1} = 10^{10.19 + 7.81 + 6.41} = 10^{24.41}$

$\beta_1 [H^+] = 10^{10.19} \times 3.98 \times 10^{-8} = 616.429$

$\beta_2 [H^+]^2 = 10^{18} \times (3.98 \times 10^{-8})^2 = 1584.04$

$\beta_3 [H^+]^3 = 10^{24.41} \times (3.98 \times 10^{-8})^3 = 162.05$

$f[A^{3-}] = 1/1 + \beta_1 [H^+] + \beta_2 [H^+]^2 + \beta_3 [H^+]^3$

$f[A^{3-}] = 1/(1 + 616.429 + 1584.04 + 162.05) = 1/2363.519 = 4.231 \times 10^{-4}$

$f[HA^{2-}] = \beta_1 [H^+] \times f[A^{3-}] = 616.429 \times 4.231 \times 10^{-4} = 0.2608$

$f[H_2A^-] = \beta_2 [H^+]^2 \times f[A^{3-}] = 1584.04 \times 4.231 \times 10^{-4} = 0.6702$

$f[H_3A] = \beta_3 [H^+]^3 \times f[A^{3-}] = 162.05 \times 4.231 \times 10^{-4} = 0.0686$

CARTESSIAN COORDINATES

Optimized geometry of quercetin at SMD/rm052x/6-311++g(d,p) level of theory in pentyl ethanoate

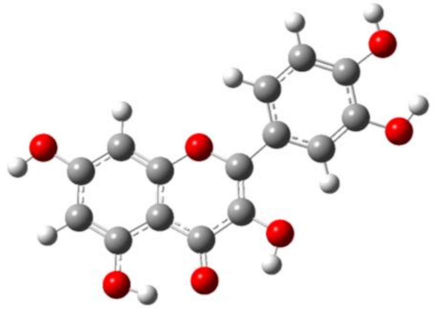

6	2.188172000	-1.460837000	-0.447334000
6	1.834972000	-0.166453000	-0.057995000
6	2.835942000	0.740119000	0.312145000
6	4.162101000	0.354941000	0.285714000
6	4.506878000	-0.939187000	-0.113083000
6	3.521830000	-1.841541000	-0.478487000
6	0.420447000	0.215625000	-0.034772000
6	-0.094345000	1.467712000	-0.115194000
6	-1.524286000	1.687980000	-0.107655000
6	-2.351278000	0.521908000	-0.020544000

6	-1.754172000	-0.740884000	0.055841000
8	-0.407642000	-0.863364000	0.050422000
6	-3.764905000	0.604752000	-0.012203000
6	-4.523226000	-0.544992000	0.079899000
6	-3.879550000	-1.784908000	0.158984000
6	-2.496188000	-1.905474000	0.146709000
8	-4.371189000	1.794525000	-0.092693000
8	-4.589326000	-2.929558000	0.253035000
8	-1.960184000	2.853301000	-0.192510000
8	0.670736000	2.576558000	-0.229029000
8	5.840447000	-1.217359000	-0.104843000
1	2.598317000	1.743322000	0.627887000
1	1.427824000	-2.170464000	-0.733981000
1	0.048568000	3.318527000	-0.298809000
1	-2.017968000	-2.870885000	0.210112000
1	-5.602955000	-0.474890000	0.088180000
1	6.003497000	-2.123277000	-0.386843000
1	-3.673131000	2.481939000	-0.152259000
1	-5.533168000	-2.736912000	0.254407000
1	3.798718000	-2.841683000	-0.788049000
8	5.126041000	1.238048000	0.650489000
1	5.984880000	0.804559000	0.588229000

Optimized geometry of quercetin 3-OH···•OOH TS at SMD/um052x/6-311++g(d,p) level of theory in pentyl ethanoate

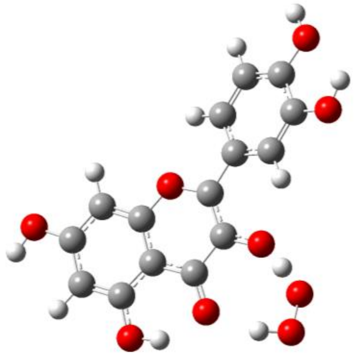

6	2.243124000	-1.775348000	0.502672000
6	1.878895000	-0.533101000	-0.038378000
6	2.881309000	0.343599000	-0.486607000
6	4.208327000	-0.016116000	-0.384330000
6	4.558777000	-1.251976000	0.171752000
6	3.576407000	-2.126628000	0.612682000
6	0.466411000	-0.198583000	-0.122101000
6	-0.081499000	1.073253000	-0.275224000
6	-1.538481000	1.226367000	-0.285804000
6	-2.320828000	0.031485000	-0.176870000
6	-1.683495000	-1.206933000	-0.044407000
8	-0.326797000	-1.281183000	-0.030490000

6	-3.739133000	0.050777000	-0.185434000
6	-4.450100000	-1.129574000	-0.069537000
6	-3.761346000	-2.336452000	0.059746000
6	-2.369890000	-2.396129000	0.073550000
8	-4.404373000	1.200792000	-0.307184000
8	-4.417116000	-3.506814000	0.178454000
8	-2.064730000	2.357433000	-0.380912000
8	0.660437000	2.134581000	-0.416781000
8	5.889230000	-1.504698000	0.230175000
1	2.638269000	1.296047000	-0.925871000
1	1.487237000	-2.459582000	0.854740000
1	0.414998000	2.893787000	0.469416000
1	-1.851016000	-3.337075000	0.174562000
1	-5.531735000	-1.100931000	-0.077465000
1	6.062930000	-2.375960000	0.603114000
1	-3.743065000	1.922500000	-0.369685000
1	-5.369644000	-3.361713000	0.155141000
1	3.859604000	-3.079218000	1.042350000
8	5.170133000	0.827627000	-0.830669000
1	6.034001000	0.421919000	-0.692534000
8	-0.777345000	4.472869000	0.584317000
1	-1.382003000	3.839166000	0.128623000
8	0.041756000	3.651044000	1.289007000

Optimized geometry of quercetin 5-OH···$^{\bullet}$OOH TS at SMD/um052x/6-311++g(d,p) level of theory in pentyl ethanoate

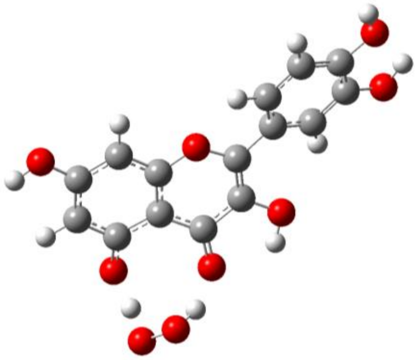

6	2.757390000	1.313214000	0.682318000
6	2.278138000	0.164342000	0.047328000
6	3.184263000	-0.776168000	-0.459858000
6	4.542656000	-0.566132000	-0.327436000
6	5.012979000	0.580224000	0.319695000
6	4.122258000	1.514941000	0.821233000
6	0.834819000	-0.029504000	-0.086720000
6	0.168263000	-1.203477000	-0.246564000
6	-1.277442000	-1.255801000	-0.274462000
6	-1.966812000	0.006472000	-0.281627000
6	-1.209531000	1.165676000	-0.095236000
8	0.134287000	1.126816000	0.015116000

6	-3.385227000	0.166054000	-0.495153000
6	-3.945692000	1.452341000	-0.399477000
6	-3.148840000	2.563611000	-0.160685000
6	-1.771907000	2.436060000	-0.018558000
8	-4.144357000	-0.813028000	-0.859933000
8	-3.657998000	3.809379000	-0.075616000
8	-1.806039000	-2.386796000	-0.255387000
8	0.795496000	-2.394256000	-0.295724000
8	6.366848000	0.687583000	0.403188000
1	2.849984000	-1.666094000	-0.968754000
1	2.069995000	2.045018000	1.078113000
1	0.086147000	-3.058958000	-0.318010000
1	-1.144653000	3.300846000	0.139594000
1	-5.012222000	1.549799000	-0.555530000
1	6.621515000	1.505996000	0.841794000
1	-4.393290000	-1.747027000	-0.070861000
1	-4.614097000	3.789170000	-0.195410000
1	4.498575000	2.398489000	1.321288000
8	5.415610000	-1.473222000	-0.831908000
1	6.314715000	-1.167707000	-0.665188000
8	-3.617340000	-2.364395000	1.632270000
1	-2.854300000	-2.542506000	1.026127000
8	-4.685790000	-2.391849000	0.795248000

Optimized geometry of quercetin 7-OH···$^•$OOH TS at SMD/um052x/6-311++g(d,p) level of theory in pentyl ethanoate

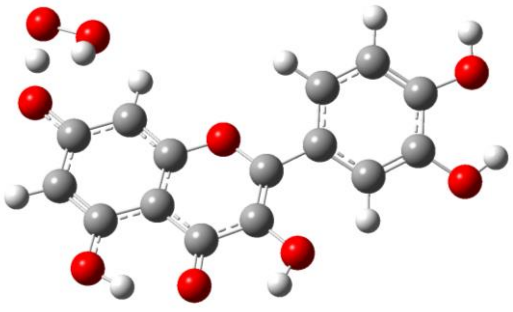

6	-2.335683000	-1.513730000	0.134056000
6	-2.198864000	-0.132050000	-0.025926000
6	-3.341086000	0.663601000	-0.183617000
6	-4.593329000	0.081922000	-0.175347000
6	-4.721322000	-1.299905000	-0.005202000
6	-3.595661000	-2.092375000	0.149140000
6	-0.859800000	0.456437000	-0.034780000
6	-0.521029000	1.745734000	0.246656000
6	0.854579000	2.181649000	0.224704000
6	1.836053000	1.183668000	-0.099608000
6	1.414608000	-0.113986000	-0.371210000
8	0.106930000	-0.446393000	-0.337688000

6	3.234331000	1.488956000	-0.156237000
6	4.141395000	0.509245000	-0.468562000
6	3.690050000	-0.799772000	-0.753423000
6	2.312437000	-1.120007000	-0.703595000
8	3.647539000	2.732012000	0.105867000
8	4.548214000	-1.733451000	-1.063642000
8	1.123260000	3.367444000	0.496285000
8	-1.428987000	2.682691000	0.580772000
8	-5.997397000	-1.770475000	-0.008708000
1	-3.271717000	1.730483000	-0.322547000
1	-1.464837000	-2.138987000	0.256056000
1	-0.925139000	3.496071000	0.748492000
1	1.969773000	-2.113779000	-0.946210000
1	5.198466000	0.727236000	-0.508958000
1	-6.013760000	-2.725486000	0.114095000
1	2.860855000	3.281706000	0.309673000
1	4.503321000	-2.586906000	-0.278583000
1	-3.706552000	-3.161423000	0.282244000
8	-5.695248000	0.856141000	-0.336089000
1	-6.479127000	0.295409000	-0.303508000
8	4.400807000	-3.232618000	0.743873000
8	3.273533000	-2.717254000	1.314229000
1	3.599442000	-2.145421000	2.027751000

Optimized geometry of quercetin 3’-OH···•OOH TS at SMD/um052x/6-311++g(d,p) level of theory in pentyl ethanoate

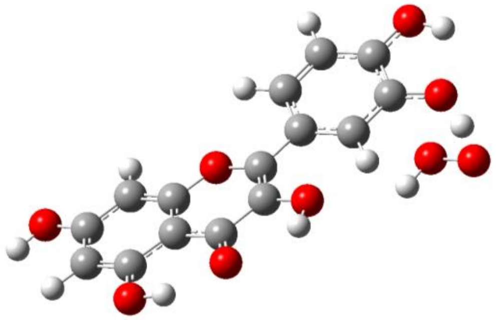

6	-1.610222000	1.639031000	-0.676995000
6	-1.325343000	0.273259000	-0.447514000
6	-2.378610000	-0.625255000	-0.343259000
6	-3.700602000	-0.175717000	-0.466978000
6	-3.961273000	1.197799000	-0.707066000
6	-2.904948000	2.096179000	-0.809751000
6	0.070927000	-0.151962000	-0.320393000
6	0.557677000	-1.414464000	-0.407219000
6	1.976857000	-1.678314000	-0.271960000
6	2.819457000	-0.544728000	-0.043531000
6	2.251867000	0.732898000	0.024337000
8	0.915522000	0.896746000	-0.111156000

6	4.221595000	-0.672545000	0.111549000
6	4.996641000	0.448965000	0.327199000
6	4.382791000	1.704978000	0.383825000
6	3.011231000	1.869234000	0.235778000
8	4.801202000	-1.876160000	0.051140000
8	5.109724000	2.822990000	0.588295000
8	2.382983000	-2.852225000	-0.366007000
8	-0.219534000	-2.495339000	-0.629048000
8	-5.221789000	1.611818000	-0.836795000
1	-2.217868000	-1.677394000	-0.168019000
1	-0.797472000	2.344529000	-0.757597000
1	0.386690000	-3.253636000	-0.667076000
1	2.557118000	2.847038000	0.287517000
1	6.067006000	0.344510000	0.446472000
1	-5.800237000	0.837900000	-0.758513000
1	4.099989000	-2.541201000	-0.114188000
1	6.042683000	2.602136000	0.682255000
1	-3.112452000	3.140943000	-0.992148000
8	-4.741889000	-0.991253000	-0.388924000
1	-4.960002000	-1.176134000	0.679190000
8	-4.214925000	0.029586000	2.166468000
1	-3.320905000	-0.311519000	2.334580000
8	-4.985395000	-1.065328000	1.963454000

Optimized geometry of quercetin 4’-OH···•OOH TS at SMD/um052x/6-311++g(d,p) level of theory in pentyl ethanoate

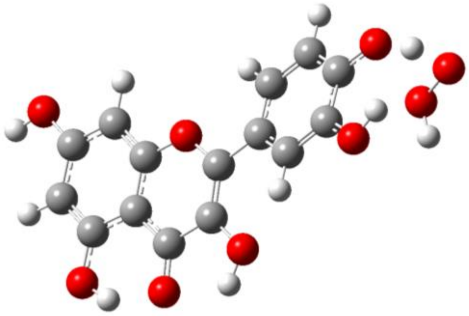

6	-1.668547000	1.377901000	-0.114852000
6	-1.253814000	0.046217000	-0.337716000
6	-2.186186000	-0.927477000	-0.700852000
6	-3.518450000	-0.575013000	-0.829430000
6	-3.945038000	0.753701000	-0.586042000
6	-2.993068000	1.722550000	-0.231713000
6	0.164176000	-0.283997000	-0.195520000
6	0.706845000	-1.513461000	-0.006654000
6	2.142385000	-1.681026000	0.146422000
6	2.934296000	-0.492503000	0.088007000
6	2.307065000	0.744269000	-0.107848000
8	0.963601000	0.819158000	-0.241242000

6	4.345262000	-0.523465000	0.218452000
6	5.070172000	0.648538000	0.151155000
6	4.396765000	1.859704000	-0.044106000
6	3.015564000	1.929506000	-0.176324000
8	4.981958000	-1.684636000	0.404678000
8	5.072307000	3.024527000	-0.113762000
8	2.595081000	-2.826711000	0.326291000
8	-0.019244000	-2.644467000	0.071305000
8	-5.233777000	1.019371000	-0.743522000
1	-1.895794000	-1.946380000	-0.897238000
1	-0.943460000	2.126570000	0.162442000
1	0.620920000	-3.359224000	0.227570000
1	2.515186000	2.873523000	-0.329007000
1	6.147385000	0.618123000	0.249369000
1	-5.717348000	1.024621000	0.239595000
1	4.311271000	-2.398280000	0.427931000
1	6.017779000	2.869040000	-0.012796000
1	-3.329036000	2.733920000	-0.048216000
8	-4.429364000	-1.496005000	-1.185339000
1	-5.276292000	-1.041062000	-1.303517000
8	-4.778839000	0.373909000	1.951643000
1	-4.809545000	-0.595122000	2.017381000
8	-5.994927000	0.737555000	1.483526000

Optimized geometry of quercetin 3-OH···$^{\bullet}OOCH_3$ TS at SMD/um052x/6-311++g(d,p) level of theory in pentyl ethanoate

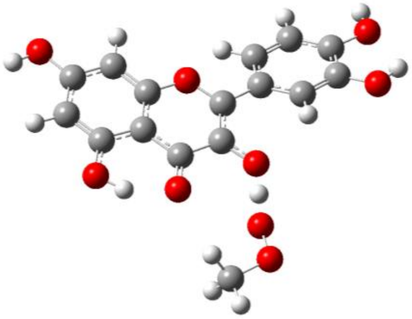

6	2.134749000	-1.964288000	0.676594000
6	1.800475000	-0.817591000	-0.056466000
6	2.817360000	-0.065230000	-0.664929000
6	4.134315000	-0.452282000	-0.532480000
6	4.457544000	-1.590605000	0.215491000
6	3.459341000	-2.341313000	0.817825000
6	0.395039000	-0.452005000	-0.180512000
6	-0.109056000	0.808139000	-0.467505000
6	-1.561467000	1.013115000	-0.523197000
6	-2.378364000	-0.147027000	-0.271413000
6	-1.783188000	-1.379933000	0.002685000
8	-0.427169000	-1.498513000	0.028358000
6	-3.792181000	-0.080342000	-0.294312000
6	-4.546105000	-1.215477000	-0.051685000

6	-3.899010000	-2.421864000	0.218136000
6	-2.510663000	-2.525735000	0.249489000
8	-4.408253000	1.076030000	-0.549316000
8	-4.596214000	-3.550890000	0.461983000
8	-2.041595000	2.131011000	-0.755534000
8	0.676162000	1.829769000	-0.688821000
8	5.781471000	-1.881123000	0.286696000
1	2.591780000	0.810771000	-1.250403000
1	1.363431000	-2.551548000	1.150198000
1	0.764960000	2.546046000	0.213379000
1	-2.023285000	-3.466473000	0.455295000
1	-5.626114000	-1.152043000	-0.072579000
1	-3.706967000	1.753053000	-0.693292000
1	-5.542288000	-3.376582000	0.408435000
8	0.832809000	3.266131000	1.182467000
8	0.640867000	4.524534000	0.730770000
6	-0.748764000	4.853827000	0.799037000
1	-0.824943000	5.870395000	0.421134000
1	-1.084330000	4.800192000	1.834234000
1	-1.310201000	4.158783000	0.174101000
1	3.721961000	-3.218843000	1.395636000
1	5.933739000	-2.679456000	0.803614000
8	5.111639000	0.267862000	-1.135549000
1	5.964374000	-0.141122000	-0.948751000

Optimized geometry of quercetin 5-OH···$^{\bullet}OOCH_3$ TS at SMD/um052x/6-311++g(d,p) level of theory in pentyl ethanoate

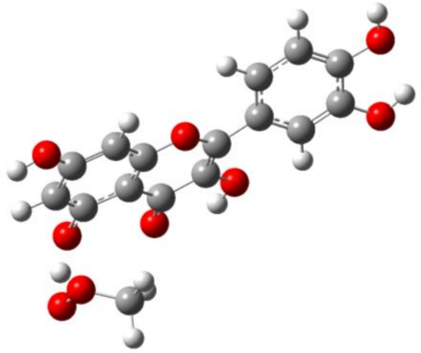

6	3.004532000	1.466710000	-0.234478000
6	2.533221000	0.158383000	-0.100554000
6	3.429776000	-0.868405000	0.220620000
6	4.770653000	-0.585705000	0.396096000
6	5.234709000	0.724412000	0.251429000
6	4.353410000	1.744953000	-0.062790000
6	1.106030000	-0.114275000	-0.290037000
6	0.517875000	-1.290078000	-0.607534000
6	-0.928214000	-1.417645000	-0.808941000
6	-1.685574000	-0.190135000	-0.643794000
6	-1.000374000	0.972759000	-0.302676000
8	0.335004000	1.001560000	-0.139859000
6	-3.108228000	-0.089341000	-0.804845000

6	-3.742776000	1.150219000	-0.596879000
6	-3.007157000	2.278456000	-0.231511000
6	-1.636616000	2.202572000	-0.086772000
8	-3.825944000	-1.119608000	-1.133553000
8	-3.594572000	3.474765000	-0.012242000
8	-1.358646000	-2.529227000	-1.103200000
8	1.207811000	-2.431900000	-0.788577000
8	6.573215000	0.893337000	0.442873000
1	3.099014000	-1.887490000	0.341377000
1	2.326144000	2.268684000	-0.481353000
1	0.531820000	-3.090255000	-1.031801000
1	-1.054626000	3.071166000	0.185110000
1	-4.815447000	1.201081000	-0.731881000
1	6.823687000	1.815350000	0.325368000
1	-4.576403000	-1.357545000	-0.255218000
1	-4.548766000	3.404606000	-0.126354000
1	4.722833000	2.757009000	-0.173090000
8	5.632955000	-1.584280000	0.713928000
1	6.519655000	-1.214482000	0.795598000
8	-4.523995000	-0.487458000	1.540422000
8	-5.111579000	-1.467441000	0.810862000
6	-3.497288000	-1.050743000	2.368916000
1	-2.843533000	-1.677568000	1.765244000
1	-2.959226000	-0.202569000	2.785429000
1	-3.963023000	-1.638793000	3.158830000

Optimized geometry of quercetin 7-OH···•OOCH$_3$ TS at SMD/um052x/6-311++g(d,p) level of theory in pentyl ethanoate

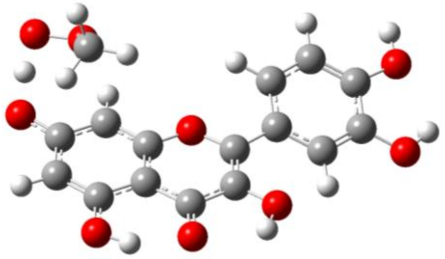

6	-2.437602000	-1.501823000	-0.053118000
6	-2.333163000	-0.108113000	-0.076757000
6	-3.496842000	0.672098000	-0.102129000
6	-4.736390000	0.063859000	-0.097836000
6	-4.831407000	-1.330533000	-0.062362000
6	-3.684884000	-2.107800000	-0.040885000
6	-1.006519000	0.508620000	-0.089719000
6	-0.678860000	1.781380000	0.268449000
6	0.687141000	2.246853000	0.217201000
6	1.670820000	1.294165000	-0.212509000
6	1.263669000	0.009063000	-0.549447000
8	-0.034907000	-0.350988000	-0.489957000
6	3.061659000	1.632310000	-0.312033000
6	3.975017000	0.691666000	-0.709575000
6	3.543291000	-0.615992000	-1.033948000

6	2.169688000	-0.959242000	-0.970891000
8	3.456951000	2.868688000	0.002138000
8	4.406900000	-1.534292000	-1.354361000
8	0.944378000	3.419335000	0.552870000
8	-1.586802000	2.676494000	0.702735000
8	-6.098001000	-1.827205000	-0.055325000
1	-3.454910000	1.748746000	-0.135974000
1	-1.550363000	-2.116068000	-0.037743000
1	-1.089973000	3.488488000	0.896800000
1	1.835941000	-1.939909000	-1.271082000
1	5.026636000	0.931940000	-0.765034000
1	-6.090974000	-2.790123000	-0.047413000
1	2.667646000	3.384817000	0.274207000
1	4.334262000	-2.413733000	-0.538543000
1	-3.768747000	-3.187159000	-0.014726000
8	-5.857608000	0.825791000	-0.132162000
1	-6.629148000	0.247557000	-0.147280000
8	4.177029000	-3.053108000	0.442245000
8	3.030479000	-2.536287000	0.959972000
6	3.337893000	-1.721488000	2.099834000
1	4.055327000	-0.951887000	1.820914000
1	2.391862000	-1.283653000	2.409376000
1	3.744018000	-2.356580000	2.885603000

Optimized geometry of quercetin 3'-OH···$^{\bullet}OOCH_3$ TS at SMD/um052x/6-311++g(d,p) level of theory in pentyl ethanoate

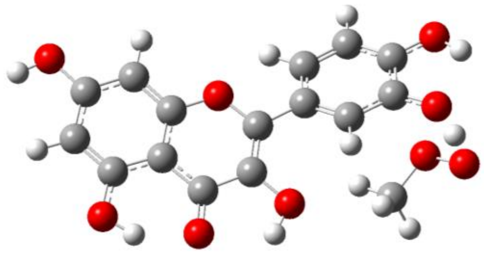

6	-1.373233000	1.808009000	0.378374000
6	-1.096128000	0.576006000	-0.258932000
6	-2.121077000	-0.111498000	-0.886086000
6	-3.423659000	0.406280000	-0.879313000
6	-3.679266000	1.646328000	-0.233317000
6	-2.646043000	2.336645000	0.394974000
6	0.269882000	0.050088000	-0.231886000
6	0.634495000	-1.253225000	-0.291237000
6	2.028251000	-1.640819000	-0.211723000
6	2.977885000	-0.578410000	-0.072148000
6	2.529505000	0.746905000	-0.017295000
8	1.207746000	1.028349000	-0.098669000
6	4.369388000	-0.826165000	0.023407000

6	5.250786000	0.227399000	0.159724000
6	4.754003000	1.534581000	0.200912000
6	3.396285000	1.816176000	0.116289000
8	4.837678000	-2.078379000	-0.018006000
8	5.586312000	2.588782000	0.326788000
8	2.321926000	-2.850843000	-0.251589000
8	-0.255337000	-2.264157000	-0.394796000
8	-4.917006000	2.139464000	-0.237178000
1	-1.950907000	-1.047362000	-1.395410000
1	-0.573341000	2.342675000	0.869230000
1	0.272056000	-3.079908000	-0.386724000
1	3.032102000	2.831488000	0.151572000
1	6.312449000	0.032002000	0.233220000
1	-5.477349000	1.539595000	-0.752503000
1	4.072924000	-2.684398000	-0.111502000
1	6.499665000	2.287829000	0.384550000
1	-2.856709000	3.274199000	0.889410000
8	-4.431556000	-0.199526000	-1.480404000
1	-4.982243000	-0.815991000	-0.700024000
8	-4.522031000	-0.724245000	1.256732000
8	-5.369905000	-1.313294000	0.381929000
6	-3.548221000	-1.674990000	1.714802000
1	-3.076697000	-2.162531000	0.864482000
1	-2.824528000	-1.100189000	2.287244000
1	-4.047161000	-2.406478000	2.348703000

Optimized geometry of quercetin 4’-OH···•OOCH$_3$ TS at SMD/um052x/6-311++g(d,p) level of theory in pentyl ethanoate

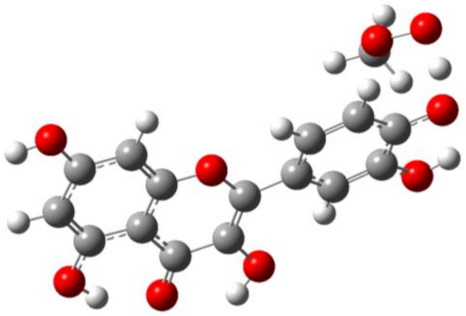

6	1.470410000	-0.998122000	-1.051549000
6	1.042796000	0.244584000	-0.529776000
6	1.978636000	1.243004000	-0.245591000
6	3.319921000	0.998993000	-0.478366000
6	3.758594000	-0.245792000	-0.998291000
6	2.801622000	-1.234670000	-1.287756000
6	-0.384496000	0.454474000	-0.292424000
6	-1.023608000	1.635305000	-0.087502000
6	-2.461903000	1.681918000	0.114903000
6	-3.152826000	0.431576000	0.088628000
6	-2.431801000	-0.748899000	-0.130487000
8	-1.093210000	-0.710406000	-0.314684000
6	-4.554254000	0.344977000	0.278762000

6	-5.178990000	-0.885062000	0.253109000
6	-4.414842000	-2.036432000	0.032782000
6	-3.039945000	-1.990259000	-0.162749000
8	-5.278737000	1.449708000	0.485855000
8	-4.991224000	-3.255160000	0.000960000
8	-3.004901000	2.788611000	0.290671000
8	-0.400570000	2.828280000	-0.068519000
8	5.051984000	-0.395330000	-1.205618000
1	1.686566000	2.197936000	0.157783000
1	0.744618000	-1.765100000	-1.270384000
1	-1.099305000	3.488521000	0.077549000
1	-2.470021000	-2.891245000	-0.331261000
1	-6.248991000	-0.944122000	0.401965000
1	5.455391000	-1.073703000	-0.394406000
1	-4.671742000	2.218549000	0.468313000
1	-5.940380000	-3.179540000	0.148176000
1	3.142393000	-2.180446000	-1.686552000
8	4.239215000	1.938530000	-0.202396000
1	5.097799000	1.594872000	-0.493057000
8	4.437402000	-1.437297000	1.312270000
8	5.630022000	-1.642878000	0.712254000
6	4.571873000	-0.449374000	2.341336000
1	3.569735000	-0.300809000	2.735360000
1	5.241450000	-0.828635000	3.111720000
1	4.965512000	0.473931000	1.918280000

Optimized geometry of rooperol at SMD/rm052x/6-311++g(d,p) level of theory in pentyl ethanoate

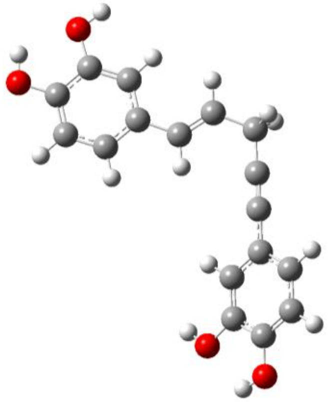

8	-4.460263000	-2.538429000	0.835829000
8	-6.647015000	-1.498265000	-0.271774000
6	0.173225000	3.104965000	0.231430000
6	-3.224357000	0.829378000	-0.024403000
6	-3.220858000	-0.481786000	0.470387000
6	-4.363825000	-1.252045000	0.383990000
6	-4.388570000	1.341316000	-0.600256000
6	-5.528491000	-0.736160000	-0.192495000
6	-5.533185000	0.559594000	-0.682500000
6	-1.039390000	2.296299000	0.137779000
6	-2.038880000	1.628125000	0.062071000

1	-3.637266000	-2.817698000	1.249107000
1	-6.462877000	-2.359529000	0.120955000
1	0.167457000	3.847318000	-0.571891000
1	0.150346000	3.674921000	1.164774000
1	-2.324197000	-0.891139000	0.918832000
1	-4.394689000	2.351553000	-0.983331000
1	-6.440930000	0.945817000	-1.125170000
6	1.461229000	2.323573000	0.168767000
6	1.551942000	1.004053000	0.013458000
1	2.351202000	2.936238000	0.257615000
1	0.635233000	0.431879000	-0.087481000
6	2.785791000	0.204946000	-0.050418000
6	2.684779000	-1.155611000	-0.340607000
6	4.061219000	0.744149000	0.167333000
6	3.813975000	-1.963771000	-0.426019000
1	1.707650000	-1.589267000	-0.508795000
6	5.180987000	-0.058099000	0.081718000
1	4.191145000	1.791557000	0.410228000
6	5.068040000	-1.420614000	-0.216739000
1	3.731699000	-3.017481000	-0.655046000
8	6.177991000	-2.198911000	-0.296004000
1	6.952286000	-1.647962000	-0.133990000
8	6.459671000	0.385848000	0.280450000
1	6.468087000	1.331987000	0.455213000

Optimized geometry of rooperol 3’-OH···•OOH TS at SMD/um052x/6-311++g(d,p) level of theory in pentyl ethanoate

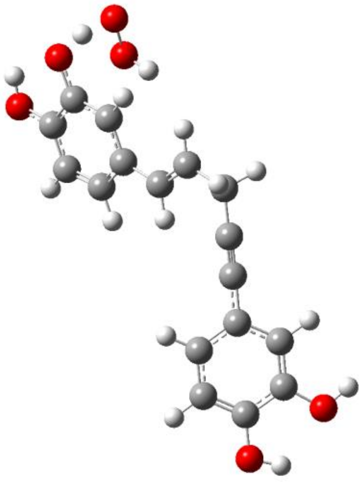

8	-6.997248000	0.406262000	1.481573000
8	-7.137363000	-1.895195000	0.155492000
6	-0.427341000	2.802701000	-0.754720000
6	-3.767358000	0.475089000	-0.299557000
6	-4.808483000	0.887731000	0.542940000
6	-5.928759000	0.093651000	0.689040000
6	-3.876886000	-0.738163000	-0.982044000
6	-6.034607000	-1.121783000	0.005565000
6	-5.005842000	-1.531373000	-0.826730000
6	-1.622706000	1.980386000	-0.588877000

6	-2.604139000	1.295169000	-0.455097000
1	-6.851575000	1.239944000	1.939848000
1	-7.742893000	-1.457166000	0.764952000
1	-0.474251000	3.314893000	-1.720214000
1	-0.429875000	3.592981000	0.001440000
1	-4.734601000	1.826430000	1.077480000
1	-3.076950000	-1.057182000	-1.634776000
1	-5.102034000	-2.473132000	-1.349341000
6	0.874535000	2.049868000	-0.669267000
6	0.989257000	0.740621000	-0.451645000
1	1.752835000	2.671207000	-0.802586000
1	0.085827000	0.156906000	-0.309799000
6	2.243662000	-0.021637000	-0.366411000
6	2.169150000	-1.377402000	0.024820000
6	3.492150000	0.511223000	-0.652273000
6	3.290849000	-2.175197000	0.143472000
1	1.196695000	-1.800420000	0.241269000
6	4.644773000	-0.273732000	-0.532000000
1	3.617666000	1.534052000	-0.981757000
6	4.539950000	-1.631025000	-0.131874000
1	3.216310000	-3.210031000	0.446505000
8	5.647414000	-2.373413000	-0.033824000
1	6.398271000	-1.816606000	-0.289005000
8	5.862660000	0.189745000	-0.797437000
1	6.192076000	0.767443000	0.064085000
8	6.282364000	1.227085000	1.299924000
8	5.391406000	0.443829000	1.951471000
1	4.549393000	0.927415000	1.922260000

Optimized geometry of rooperol 4’-OH···•OOH TS at SMD/um052x/6-311++g(d,p) level of theory in pentyl ethanoate

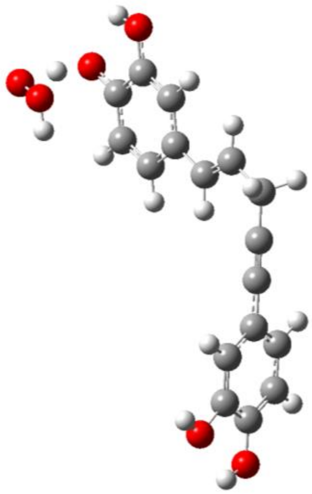

8	6.235555000	-1.456812000	1.740547000
8	7.024020000	-1.882569000	-0.764627000
6	0.643140000	3.146281000	0.619586000
6	3.817998000	0.653650000	-0.078349000
6	4.469348000	0.036212000	0.998254000
6	5.536069000	-0.808188000	0.762274000
6	4.257274000	0.404965000	-1.379992000
6	5.974817000	-1.054079000	-0.542531000
6	5.330793000	-0.445961000	-1.607612000
6	1.778397000	2.263014000	0.369102000

6	2.711443000	1.529295000	0.164570000
1	5.888165000	-1.239342000	2.611252000
1	7.348140000	-2.206523000	0.084088000
1	0.690104000	3.505611000	1.651481000
1	0.733366000	4.037450000	-0.008512000
1	4.138619000	0.221241000	2.012433000
1	3.757714000	0.879989000	-2.212204000
1	5.680430000	-0.645759000	-2.611070000
6	-0.708100000	2.526537000	0.385885000
6	-0.916691000	1.279700000	-0.040014000
1	-1.539707000	3.189998000	0.591918000
1	-0.058330000	0.648739000	-0.243207000
6	-2.221180000	0.655185000	-0.276712000
6	-2.235223000	-0.665248000	-0.771474000
6	-3.433764000	1.307894000	-0.036021000
6	-3.423565000	-1.316540000	-1.022938000
1	-1.294665000	-1.165078000	-0.960693000
6	-4.629396000	0.659135000	-0.279403000
1	-3.469675000	2.318449000	0.344770000
6	-4.642091000	-0.668420000	-0.775599000
1	-3.444743000	-2.325510000	-1.413976000
8	-5.826567000	-1.229724000	-1.010820000
1	-6.036902000	-1.921209000	-0.217116000
8	-5.797829000	1.280325000	-0.052046000
1	-6.507258000	0.674525000	-0.309816000
8	-5.084677000	-1.777433000	1.637777000
1	-4.223437000	-2.196664000	1.473149000
8	-5.990182000	-2.533352000	0.983338000

Optimized geometry of rooperol 3’’-OH···•OOH TS at SMD/um052x/6-311++g(d,p) level of theory in pentyl ethanoate

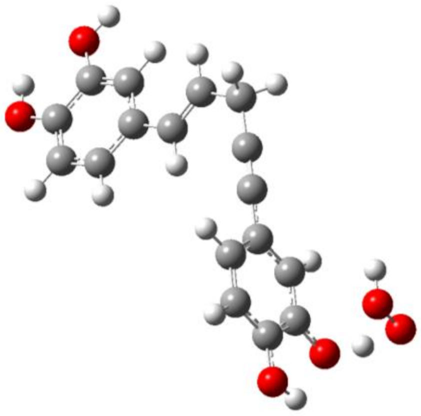

8	4.850872000	-1.157768000	-1.673905000
8	5.727763000	-2.137242000	0.664456000
6	-0.797660000	2.874300000	0.589401000
6	2.458951000	0.392926000	0.596623000
6	3.093807000	0.057268000	-0.589525000
6	4.207344000	-0.793967000	-0.573052000
6	2.942187000	-0.137174000	1.815008000
6	4.673195000	-1.318516000	0.660429000
6	4.031160000	-0.984167000	1.848906000
6	0.368696000	1.996848000	0.595901000
6	1.323073000	1.261940000	0.595260000
1	5.522256000	-0.336201000	-1.961993000

1	6.008040000	-2.264583000	-0.254418000
1	-0.824412000	3.435260000	1.527595000
1	-0.674619000	3.618787000	-0.202171000
1	2.744022000	0.437541000	-1.540000000
1	2.439494000	0.128274000	2.734304000
1	4.397879000	-1.389013000	2.781521000
6	-2.117197000	2.168457000	0.399736000
6	-2.274814000	0.852632000	0.268867000
1	-2.973121000	2.833878000	0.387528000
1	-1.397195000	0.215830000	0.314332000
6	-3.549207000	0.139284000	0.086254000
6	-3.573022000	-1.245160000	0.255899000
6	-4.743794000	0.789446000	-0.251997000
6	-4.751958000	-1.969737000	0.114491000
1	-2.657013000	-1.763711000	0.507372000
6	-5.912599000	0.068590000	-0.395093000
1	-4.766314000	1.858792000	-0.422991000
6	-5.927977000	-1.317927000	-0.209493000
1	-4.769354000	-3.042467000	0.250928000
8	-7.083646000	-2.015395000	-0.357205000
1	-7.786420000	-1.398369000	-0.591086000
8	-7.119383000	0.619547000	-0.730359000
1	-7.035036000	1.568811000	-0.862991000
8	6.089284000	1.116781000	-0.612104000
1	5.350026000	1.747082000	-0.615811000
8	6.250192000	0.741555000	-1.903108000

Optimized geometry of rooperol 4’’-OH···•OOH TS at SMD/um052x/6-311++g(d,p) level of theory in pentyl ethanoate

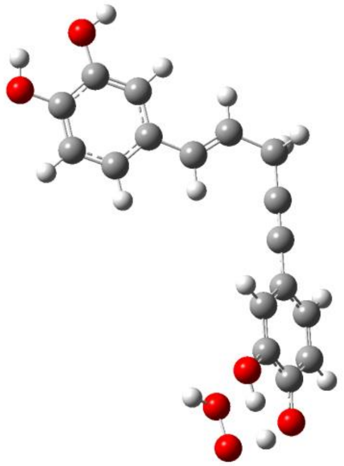

8	-4.052296000	-1.463218000	-1.956939000
8	-6.113831000	-0.711296000	-0.428313000
6	1.027117000	3.289195000	0.110858000
6	-2.541295000	1.308649000	-0.159020000
6	-2.680510000	0.239114000	-1.046136000
6	-3.890744000	-0.428491000	-1.114882000
6	-3.622086000	1.711660000	0.654271000
6	-4.976791000	-0.037937000	-0.292449000
6	-4.822732000	1.045460000	0.586537000
6	-0.246105000	2.584664000	0.011187000

6	-1.296736000	2.000531000	-0.072583000
1	-4.981247000	-1.733924000	-1.912229000
1	-6.187333000	-1.443862000	0.370630000
1	1.099904000	4.001057000	-0.716930000
1	1.026746000	3.890442000	1.024057000
1	-1.859652000	-0.067600000	-1.678551000
1	-3.491303000	2.543201000	1.331095000
1	-5.663252000	1.331193000	1.204518000
6	2.244291000	2.399180000	0.104759000
6	2.226168000	1.070410000	0.020842000
1	3.181190000	2.939371000	0.178471000
1	1.267671000	0.566536000	-0.051493000
6	3.393239000	0.174627000	0.016515000
6	3.178934000	-1.200197000	-0.077793000
6	4.713486000	0.637488000	0.105037000
6	4.240404000	-2.099718000	-0.084931000
1	2.165782000	-1.574034000	-0.145507000
6	5.765666000	-0.255543000	0.097779000
1	4.929456000	1.696145000	0.180283000
6	5.538999000	-1.633244000	0.002781000
1	4.070113000	-3.165252000	-0.157393000
8	6.584793000	-2.498846000	-0.002474000
1	7.402516000	-1.992876000	0.067258000
8	7.082231000	0.106738000	0.179149000
1	7.169828000	1.062549000	0.246982000
8	-4.614579000	-1.893886000	1.634528000
1	-3.989814000	-2.505654000	1.210835000
8	-5.842373000	-2.337290000	1.282966000

Optimized geometry of rooperol allylic H···•OOH TS (in back of molecular plane) at SMD/um052x/6-311++g(d,p) level of theory in pentyl ethanoate

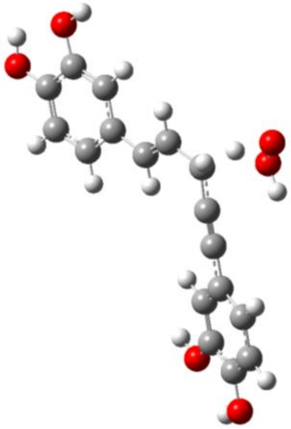

8	-4.439875000	-2.823585000	0.913038000
8	-6.722285000	-1.803187000	0.013904000
6	0.195800000	2.528424000	-0.864834000
6	-3.236764000	0.383078000	-0.456865000
6	-3.199844000	-0.872450000	0.167905000
6	-4.365343000	-1.594634000	0.322367000
6	-4.456271000	0.889577000	-0.914073000
6	-5.585107000	-1.085590000	-0.138574000
6	-5.622995000	0.155914000	-0.753208000
6	-1.006760000	1.751756000	-0.773380000
6	-2.031424000	1.128740000	-0.620545000
1	-3.566966000	-3.134041000	1.174051000

1	-6.507454000	-2.638834000	0.445172000
1	0.161452000	3.274854000	-1.657653000
1	0.131664000	3.239285000	0.141565000
1	-2.259773000	-1.272669000	0.526917000
1	-4.485749000	1.855753000	-1.396644000
1	-6.572981000	0.534298000	-1.104639000
6	1.487159000	1.825290000	-0.762835000
6	1.626474000	0.542141000	-0.397440000
1	2.355736000	2.437490000	-0.973109000
1	0.730838000	-0.043517000	-0.217860000
6	2.886959000	-0.182893000	-0.236038000
6	2.846328000	-1.565550000	-0.044383000
6	4.138469000	0.450641000	-0.264322000
6	4.012026000	-2.307795000	0.102147000
1	1.888549000	-2.067920000	-0.014522000
6	5.294133000	-0.287264000	-0.117572000
1	4.215654000	1.523659000	-0.389937000
6	5.241737000	-1.674852000	0.064890000
1	3.980542000	-3.378681000	0.248236000
8	6.384930000	-2.388802000	0.210674000
1	7.135425000	-1.785821000	0.154711000
8	6.553452000	0.244882000	-0.127867000
1	6.519705000	1.198772000	-0.250861000
8	-0.183639000	3.755479000	1.351461000
8	-0.541529000	2.666732000	2.082248000
1	-1.482539000	2.538011000	1.887474000

Optimized geometry of rooperol allylic H···•OOH TS (in front of molecular plane) at SMD/um052x/6-311++g(d,p) level of theory in pentyl ethanoate

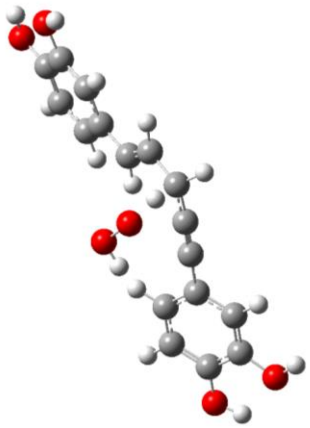

8	-6.691604000	-0.207325000	1.096519000
8	-6.469522000	-2.231491000	-0.611787000
6	0.171927000	2.346523000	0.917384000
6	-3.125413000	0.072116000	0.200602000
6	-4.347601000	0.363984000	0.823481000
6	-5.457794000	-0.406988000	0.546506000
6	-3.046946000	-0.997171000	-0.695718000
6	-5.375937000	-1.478335000	-0.350170000
6	-4.169235000	-1.766395000	-0.967872000
6	-0.989632000	1.525096000	0.727457000

6	-1.971581000	0.863704000	0.483239000
1	-6.674194000	0.522634000	1.723694000
1	-7.209676000	-1.897096000	-0.091433000
1	0.119517000	3.109381000	-0.050525000
1	0.076017000	3.040658000	1.751189000
1	-4.418701000	1.189008000	1.520785000
1	-2.105810000	-1.222655000	-1.176812000
1	-4.122133000	-2.597443000	-1.657972000
6	1.493994000	1.704106000	0.821132000
6	1.695738000	0.445273000	0.402037000
1	2.329908000	2.341349000	1.083047000
1	0.829594000	-0.167732000	0.175823000
6	2.987994000	-0.221871000	0.244674000
6	3.007332000	-1.600588000	0.021517000
6	4.211682000	0.461740000	0.309471000
6	4.204910000	-2.291605000	-0.118147000
1	2.071107000	-2.139919000	-0.038009000
6	5.398793000	-0.225279000	0.167886000
1	4.242021000	1.533901000	0.459610000
6	5.406510000	-1.609610000	-0.043865000
1	4.219656000	-3.359295000	-0.288746000
8	6.581370000	-2.271553000	-0.182488000
1	7.303398000	-1.635792000	-0.114147000
8	6.635382000	0.356667000	0.211564000
1	6.560427000	1.308612000	0.331370000
8	-0.133814000	3.636640000	-1.270167000
8	-0.248493000	2.541356000	-2.066550000
1	-1.176127000	2.271856000	-1.982281000
